# Commentary: Spatial Olfactory Learning Contributes to Place Field Formation in the Hippocampus

**DOI:** 10.3389/fnsys.2018.00008

**Published:** 2018-04-10

**Authors:** Mikhail A. Lebedev, Alexei Ossadtchi

**Affiliations:** ^1^Department of Neurobiology, Duke University, Durham, NC, United States; ^2^Center for Bioelectric Interfaces of the Institute for Cognitive Neuroscience of the National Research University Higher School of Economics, Moscow, Russia

**Keywords:** olfactory response, hippocampus, place cells, place cell remapping, scent marking, navigation

The discovery of place-representing neurons in the hippocampal formation has been recognized by the Nobel Committee as a paradigm shift in Neuroscience (Burgess, 2014). Here we call attention to an innovative paper of particular note (Zhang and Manahan-Vaughan, 2015) that added important findings to this field of study.

Zhang and Manahan-Vaughan investigated the contribution of olfactory cues to the formation of place fields in hippocampal neurons. For this purpose, they put male Wistar rats in the darkness into a 80 × 80 cm square box. Four odors (orange, vanilla, almond, and lemon) were placed into the quadrants of the arena. Chocolate crumbs were scattered across the arena to encourage exploratory behavior. The researchers observed the formation of stable place fields in the hippocampal neurons, even though visual cues were unavailable to the rats. The place fields rotated when the odor placements were rotated, and remapped when the odors were shuffled. The authors concluded that “despite the less precise nature of olfactory stimuli compared with visual stimuli, these can substitute for visual inputs to enable the acquisition of metric information about space.”

This is a significant finding because it provides insights on the role of olfaction in the formation of hippocampal representation of space, or “cognitive maps” using the terminology of O'Keefe and Nadel (1978). O'Keefe's experiments utilized a neuro-ethological approach, where neuronal responses were examined during natural animal behaviors, such as spatial exploration and foraging by unrestrained rodents (O'Keefe and Nadel, 1978). O'Keefe's key discovery was the finding of place cells in the rat hippocampus that discharged when a rat entered a particular spatial location (O'Keefe and Dostrovsky, 1971). O'Keefe and his colleagues reported that place cells responded to environmental visual cues (O'keefe and Conway, 1978). Moser et al. (2008) commented on this development, “Early on, it became apparent that place fields are strongly influenced by distal sensory cues.” Following this discovery, many studies focused on the role of vision in place field formation, and the integration of visual inputs with vestibular and proprioceptive inputs during path integration (Markus et al., 1994; Wiener et al., 1995; Arleo and Gerstner, 2000; Moser et al., 2008). The relationship of olfaction and place fields was less studied. Several recent studies employed virtual reality tasks to eliminate the contribution of olfaction entirely and to show that vision alone can generate place fields (Harvey et al., 2009; Dombeck et al., 2010; Chen et al., 2013; Domnisoru et al., 2013; Ravassard et al., 2013; Aronov and Tank, 2014; Aghajan et al., 2015). Given the predominance of studies based on visual environments, the Zhang and Manahan-Vaughan study of olfaction is a contribution to a less explored field.

While most of the Zhang and Manahan-Vaughan findings are convincing, the paper does not contain a thorough analysis of rat navigation traces. This is unfortunate because rodents generate highly structured navigation patterns (Golani et al., 1993; Drai and Golani, 2001; Benjamini et al., 2010; Yaski et al., 2011) characterized by the establishment of a home base, tendency to stay near the walls and corners, and locomotion periods intermingled with stops and turns. It seems reasonable to hypothesize that in the experiments of Zhang and Manahan-Vaughan, rats behaved differently in the odor locations compared to the other parts of the arena. For example, they could have exhibited specific sniffing patterns; additionally, they could have explored some odors longer than the others. Furthermore, since navigation goal was to obtain chocolate crumbs, rats could have developed a specific foraging strategy, such as of avoiding locations that have been already visited and seeking food at new locations. Analysis of navigation statistics is important because navigation patterns could have influenced the measurements of the neuronal place fields. For example, if the rats frequently visited some places in the arena but rarely visited the others, measurements of neuronal rates are more reliable for the former locations than for the latter. It is not entirely clear why Zhang and Manahan-Vaughan displayed neuronal place fields using a circular area although the arena had a square shape. Finally, the duration of each analyzed session was 5 min, the time that may have been insufficient for a rat to cover all places in the arena. These issues are present not only in the study of Zhang and Manahan-Vaughan; most of the studies on place cells and grid cells contain little detail on navigation traces and different behaviors exhibited by rodents during navigation, even though non-random navigation patterns are often visible in the published figures that display animal traces.

Notably, the study of Zhang and Manahan-Vaughan contains no data on the behavioral responses to different odors. Were the sniffing patterns (Welker, 1964; Clarke et al., 1970; Youngentob et al., 1987; Fonio et al., 2015) altered? Were the spatially-dependent neuronal responses phase locked to the sniffing? Obtaining answers to these questions is important because different sniffing (and postural, and locomotion) patterns in specific parts of the arena could have produced distinct neuronal responses, leading to an erroneous conclusion of the presence of a purely spatial map in the hippocampus, irrespective of the behaviors exhibited by the animals. In reality, the neuronal patterns may have represented neuronal responses to specific behaviors, not a spatial map *per se*.

Lastly, Zhang and Manahan-Vaughan do not mention scent marking behavior, which is prevalent in rodents, particularly in the male animals (Johnson, 1973; Tomlinson and Johnston, 1991; Wallace et al., 2002; Stopka and MacDonald, 2003; Hurst and Beynon, 2004; Kulvicius et al., 2008). Based on the findings of Zhang and Manahan-Vaughan, it is reasonable to suggest that odors resulting from scent marking could have contributed to the formation of hippocampal spatial maps. Such a suggestion would be consistent with O'Keefe's neuro-ethological approach because male rodents (predominantly used in studies on place fields) utilize scent marks to advertise their ownership of a territory and attract females (Roberts et al., 2010, 2012; Thonhauser et al., 2013). Additionally, rodents respond to odor cues during navigation (Lavenex and Schenk, 1998; Wallace et al., 2002; Porter et al., 2005; Khan et al., 2012), although vision dominates over olfaction in certain tasks (Small, 1901; Olton and Collison, 1979; Lavenex and Schenk, 1996; Maaswinkel and Whishaw, 1999).

Overall, Zhang and Manahan-Vaughan agreed with the conventional notion that “visuospatial contexts comprise a key element in the formation of place fields” and attributed only a secondary role to olfaction. Yet, they cited several studies showing that the contribution of olfaction could be quite significant. For example, blind rats exhibit place fields (Save et al., 1998). Additionally, stability of place fields improves when the recording box is not cleaned in between the experiments (Save et al., 2000). Zhang and Manahan-Vaughan themselves reported that hippocampal neurons exhibited place fields even when no odors were experimentally placed in the recording box. Moreover, these place fields persisted after the rats were taken out of the box and then put back while the light was off. Since this manipulation should have disoriented the rats, the fact that the place fields remained indicates that some sensory cues were left in the box, possibly scent marks spared by the cleaning procedure. Most of the labs involved in this research clean the behavioral arena in between the experimental sessions. However, their cleaning procedures may be insufficient for eliminating the scent marks entirely. For example, the following cleaning procedure is reported in the pioneering study of the entorhinal grid cells (Fyhn et al., 2004): “Each trial lasted 10 min. Before and after each trial, the rat rested on a pedestal for 5–10 min, and the floor was cleaned with a damp cloth.” This may qualify only as a partial cleaning that does not remove odors completely (Gray and Hurst, 1995). Additionally, even if the cleaning worked well, scent marks may have gradually accumulated in the arena during the 10-min experimental session.

Historically, Ramon Cajal initially proposed that the hippocampus was a structure with an essential role in olfaction (DeFelipe and Jones, 1988; Vanderwolf, 2001). Cajal's theory was, however, abandoned after Brodal questioned the existence of olfactory inputs to the hippocampus and pointed to the studies showing that hippocampal lesions did not affect behaviors conditioned by odors (Brodal, 1947). Olfactory projections to the hippocampal formation were later shown (Krettek and Price, 1977; Luskin and Price, 1983; Room et al., 1984; Schwerdtfeger et al., 1990); as well as electrophysiological responses to odors (Wilson and Steward, 1978; Vanderwolf, 1992; Biella and De Curtis, 2000; Insausti et al., 2002). Yet, only Vanderwolf continued to believe that the hippocampal formation processed odors instead of constructing a spatial map (Vanderwolf, 1992, 2001), whereas the mainstream theory described the hippocampal formation a hierarchically high region that constructs a cognitive map of space based on multiple sensory inputs (Moser and Moser, 2013). Curiously, this theory does not explain why cetaceans orient in space well (Thomas and Kastelein, 2013) despite having a small hippocampus (Patzke et al., 2015). Conspicuously, their small size of the hippocampus correlates with the absence of olfaction in these animals (Breathnach, 1960). Additionally, Jacobs (2012) proposed that rodents rely on olfaction when they navigate in space, and that olfactory regions act as a scaffold for the visual representation of space. In agreement with this view, olfactory bulbectomy severely impairs navigation in rats (van Rijzingen et al., 1995).

Based on the study of Zhang and Manahan-Vaughan and the other studies on the processing of olfactory information in the hippocampus (Shapiro et al., 1997; Save et al., 2000; Deshmukh and Bhalla, 2003; MacDonald et al., 2013), one could question the purely visual origin of the hippocampal place cells reported in some of the previous publications, particularly the ones with inadequate controls for scent marks and different navigation-related behaviors, such as sniffing (Welker, 1964; Clarke et al., 1970; Kepecs et al., 2005), whisking (Berg and Kleinfeld, 2003; Leiser and Moxon, 2007; Mitchinson et al., 2007) and locomotion (Parker and Clarke, 1990; Vásquez et al., 2002; Eilam et al., 2003), as well lacking control for technical issues, such as visual cues, forces, and even sounds produced by the electrical cable attached to the head implant and suspended over the animal. These issues could be addressed by adding appropriate controls. Thus, scent marks could be made visible in ultraviolet illumination (Desjardins et al., 1973). Additionally, traces of urinary proteins could be detected using a polyclonal antibody (Beynon and Hurst, 2004). The located scent marks could be then compared with the recordings of animal trajectories and behavioral data. Figure 1 illustrates a possible procedure that could clarify this issue.

**Figure 1:**
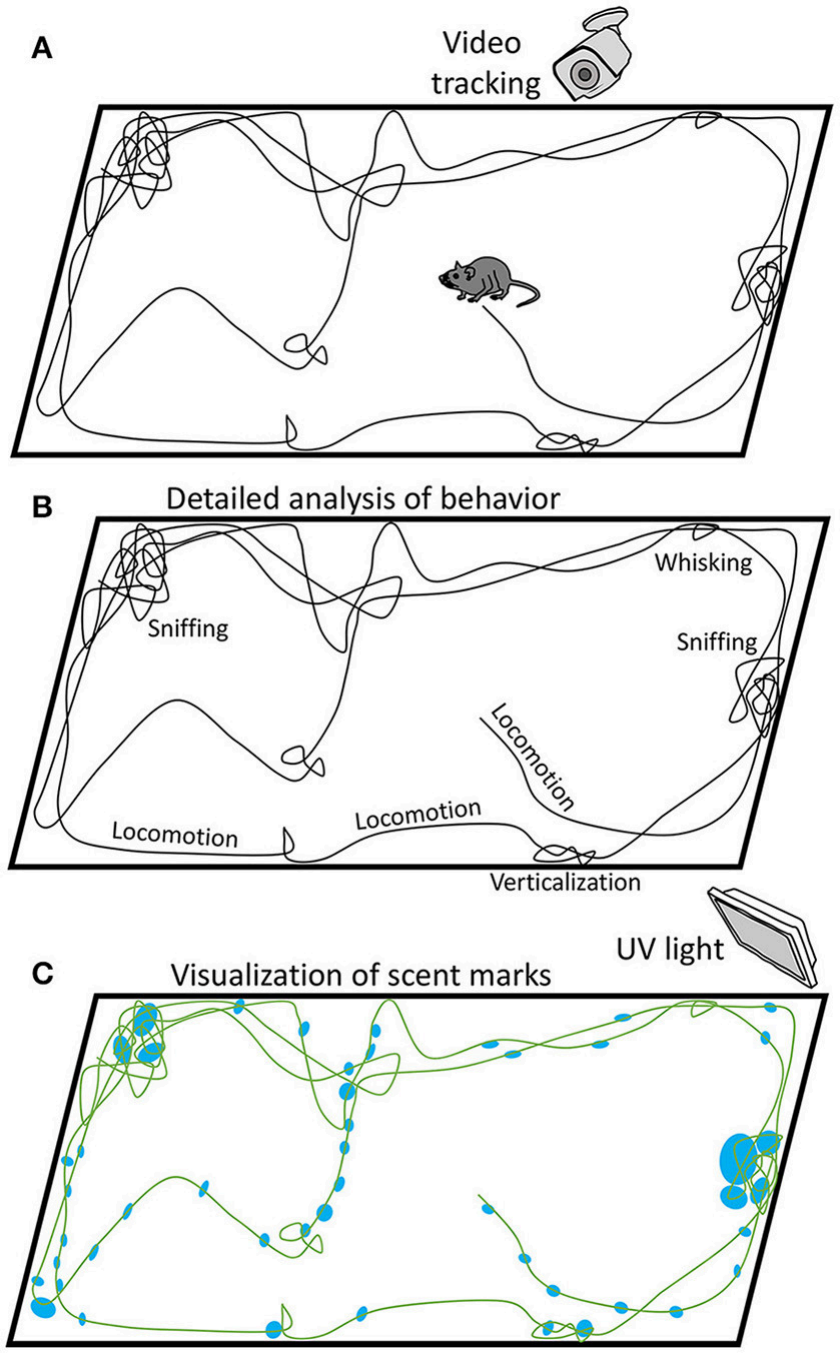
Possible approach to the analysis of different behaviors associated with rodent navigation, including scent marking. **(A)** While a rodent navigates in an arena, its position and different behaviors are video recorded. **(B)** Behaviors, such as locomotion, sniffing, whisking and verticalization, are analyzed based on video recordings. Maps of animal position and velocity are analyzed, as well. **(C)** Scent marks are visualized under UV light and compared with the navigation traces.

A point of view should be acknowledged, stating that the contribution of olfaction is no longer a concern for the studies of rodent navigation in virtual environments, where odors left on the physical treadmill do not match virtual visual stimuli (Harvey et al., 2009; Dombeck et al., 2010; Chen et al., 2013; Domnisoru et al., 2013; Ravassard et al., 2013; Aronov and Tank, 2014; Aghajan et al., 2015). Yet, a closer look at these studies reveals several issues. For example, Figure 4A in the study of Aronov and Tank (2014) shows a clearly non-uniform occupancy map for the virtual arena (e.g., zoom in their panel marked “Cell 2”). The map indicates that the rats visited different places with different probability and possibly behaved (whisked, sniffed, walked) differently depending on their location in the virtual environment. Similar non-uniform occupancy maps can be found in Figures 2A–C of Domnisoru et al. (2013). Furthermore, Movie S1 of Aronov and Tank shows that their rat sniffed the physical treadmill from time to time, i.e., exhibited an olfactory-related behavior. It seems reasonable to assume that the visual virtual stimuli may have triggered sniffing, which in turn may have modulated the activity of hippocampal neurons. The absence of any consistent smell in the virtual places may have facilitated the sniffing-induced neural responses rather than attenuating them. The modulation of hippocampal activity by sniffing has been known since O'Keefe's study of “misplace units” that responded to exploratory sniffing of places where a familiar object was missing, or a new object was found (O'Keefe, 1976). Additionally, sniffing activates the olfactory system even when no odors are present (Adrian, 1942; MacRides and Chorover, 1972). After all, rodent olfactory-related behaviors may not be easy to rule out, even in the virtual environments that uncouple visually-defined places from the odors present in the real environment.

In addition to visual virtual environments, tactile (Sofroniew et al., 2015; Thurley and Ayaz, 2017) and olfactory-based (Radvansky and Dombeck, 2018) environments have been developed. While these studies help to elucidate the specific roles of different modalities in the formation of place fields, our comments still apply regarding the need for a more thorough analysis of different behaviors associated with rodent navigation.

In conclusion, the work of Zhang and Manahan-Vaughan not only contributes important findings on the formation of place fields based on olfactory inputs, but also instigates critical thinking regarding the methodological approaches in this field and the theories of hippocampal function.

## Author contributions

All authors listed have made a substantial, direct and intellectual contribution to the work, and approved it for publication.

### Conflict of interest statement

The authors declare that the research was conducted in the absence of any commercial or financial relationships that could be construed as a potential conflict of interest.
